# Advances in multi-omics integrated analysis methods based on the gut microbiome and their applications

**DOI:** 10.3389/fmicb.2024.1509117

**Published:** 2025-01-03

**Authors:** Dongdong Duan, Mingyu Wang, Jinyi Han, Mengyu Li, Zhenyu Wang, Shenping Zhou, Wenshui Xin, Xinjian Li

**Affiliations:** ^1^Sanya Institute, Hainan Academy of Agricultural, Sanya, China; ^2^College of Animal Sciences and Technology, Henan Agricultural University, Zhengzhou, China

**Keywords:** multi-omics integrated analysis, intestinal microbiota, host genome, metabolome, MGWAS

## Abstract

The gut microbiota actually shares the host’s physical space and affects the host’s physiological functions and health indicators through a complex network of interactions with the host. However, its role as a determinant of host health and disease is often underestimated. With the emergence of new technologies including next-generation sequencing (NGS) and advanced techniques such as microbial community sequencing, people have begun to explore the interaction mechanisms between microorganisms and hosts at various omics levels such as genomics, transcriptomics, metabolomics, and proteomics. With the enrichment of multi-omics integrated analysis methods based on the microbiome, an increasing number of complex statistical analysis methods have also been proposed. In this review, we summarized the multi-omics research analysis methods currently used to study the interaction between the microbiome and the host. We analyzed the advantages and limitations of various methods and briefly introduced their application progress.

## 1 Introduction

Metabolome gut microbiome is a complex ecosystem composed of thousands of bacteria, viruses, fungi, and protozoa, which have certain regulatory effects on the host’s metabolism and immune system along with many other physiological functions. The gut microbiota is typically transmitted from the mother to the infant at birth through processes such as delivery, breastfeeding, and skin contact, helping to establish the initial colonization of beneficial microorganisms in the infant’s gut. As the infant grows, the gut microbiota continues to be influenced by environmental factors, including the host’s nutrition, lifestyle, immune status, and medication, leading to further changes in its composition (Jandhyala et al., 2015). Changes in the gut microbiota will then affect the host’s health status, leading to the occurrence of diseases including colorectal cancer (Rebersek, 2021), inflammatory bowel disease (Santana et al., 2022), obesity, and depression (Breton et al., 2022; Cheung et al., 2019).

Although the gut microbiota has a significant impact on host health, research progress on the gut microbiota has been relatively slow due to technical limitations. In recent years, with advancements in sequencing technology, research on the gut microbiota has shown explosive growth, and researchers have gained a more intuitive understanding of the composition of the gut microbiota.

The current research on the gut microbiota is gradually revealing the complex interaction relationship between microorganisms and their hosts. For example, the interactions among gut microbiota change with the alteration of its host’s disease status (Cao et al., 2022); metabolomics combined with metagenomics analysis results show that gut microbiota has the ability to metabolize drugs (Zimmermann et al., 2019); combined analysis of microbiome and host transcriptome also indicates that gut microbiota can regulate the expression of host genes (Richards et al., 2019). Therefore, in order to further explore the interaction between the gut microbiota and the host, multi-omics integrated analysis based on the microbiome has been widely conducted. This article provides a review of the multi-omics integrated analysis methods.

## 2 Microbiome data analysis methods and their limitations

### 2.1 Microbiome data analysis methods

Current microbiota analysis methods mainly include shotgun sequencing and 16S rRNA amplicon sequencing (Sharpton, 2014). In shotgun sequencing, researchers first extract DNA from the sample and sequence it. Then, computational methods are used to align the reads with a reference genome or marker genes to infer the abundance of microbial communities in the sample (Sharpton, 2014). In 16S rRNA amplicon sequencing, researchers only amplify and sequence a fragment of the 16S rRNA gene from the bacterial genome in the sample. This sequencing method uses conserved regions of the 16S rRNA gene as the target for PCR primers, with variable regions used for the classification of microbial communities in the sample.

Current microbial sequencing technologies are primarily divided into short-read sequencing (e.g., Illumina) and long-read sequencing (e.g., Nanopore), each with distinct characteristics and applications. Short-read sequencing, known for its high throughput, low cost, and accuracy, is widely used in large-scale microbial genome sequencing projects, especially when rapid sequencing of a large number of samples is required (Xia et al., 2023). This technology generates abundant short sequences, which are valuable for studying microbial communities and their functions. On the other hand, long-read sequencing, which provides longer sequence lengths, is particularly useful for analyzing complex genomic regions, such as structural variations and repetitive sequences, enabling more accurate microbial genome analysis. Furthermore, long-read sequencing allows for real-time data output, making it suitable for studies that require rapid feedback. However, both sequencing methods have their limitations. Short-read sequencing, due to its shorter read lengths, faces challenges in sequence assembly, particularly in complex genomic regions, and struggles with identifying repetitive sequences and structural variations, often requiring additional validation. In contrast, long-read sequencing tends to be more expensive and has a higher error rate. Therefore, both short-read and long-read sequencing technologies have their respective advantages and drawbacks, and the choice between them should be based on the specific research needs and budget constraints (Pervez et al., 2022).

In addition to sequencing methods, microbial taxonomic annotation is a key component of microbial sequencing analysis. There are numerous taxonomic annotation tools available, each with its own strengths and limitations, making them suitable for different applications. QIIME 2 (Quantitative Insights Into Microbial Ecology 2), for example, offers a wide range of functions, including data preprocessing, sequence filtering, clustering, and visualization. It can also be extended through the installation of plugins, making it widely used in 16S/18S rRNA sequence analysis, microbial community analysis, and metagenomic analysis. However, QIIME 2 requires command-line operation, which necessitates some programming skills, and it demands significant computational resources. Another widely used tool is MOTHUR, an open-source, extensible platform that supports a variety of functions, including sequence quality control, clustering, classification, and species abundance analysis. While MOTHUR offers a more comprehensive feature set, it requires higher levels of computational and biological expertise, which makes it more complex to use. As a result, its user base is smaller than that of QIIME 2, and it is primarily applied in 16S rRNA sequence processing and ecological studies of microbial communities. Kraken is another commonly used taxonomic tool based on k-mer classification. It is fast, especially well-suited for large-scale datasets, and offers higher accuracy compared to traditional methods. However, Kraken requires more memory for data processing, and its downstream analysis capabilities are not as comprehensive as those of QIIME 2 and MOTHUR, limiting its broader application (Lu and Salzberg, 2020). Kraken is primarily used for the rapid classification of metagenomic and metatranscriptomic data, particularly in large-scale datasets. BLAST (Basic Local Alignment Search Tool) is a classic sequence alignment tool that provides high-precision sequence similarity searches and taxonomic annotation, with a frequently updated database. However, BLAST has limitations for large-scale data analysis, as it only performs alignment and does not offer community analysis or functional predictions. Additionally, due to the time-consuming nature of its alignment process, BLAST is commonly used for precise alignment of individual gene sequences in smaller datasets. MetaPhlAn (Metagenomic Phylogenetic Analysis) is a tool specifically designed for metagenomic sequencing, known for its high accuracy and targeted approach. However, it is limited to metagenomic data analysis and does not perform well with 16S rRNA data, making it unsuitable for studies that require broader data types (Bokulich et al., 2018). MetaPhlAn is mainly used for detailed analysis of microbial community composition in metagenomic datasets. Tools like RDP Classifier, USEARCH/UPARSE, and SILVA are specifically designed for 16S rRNA sequencing data and are not applicable to metagenomic sequencing (Zou et al., 2023). These tools are often used for the classification of 16S/18S rRNA gene sequences and are particularly useful for microbial community research. In conclusion, each tool and database has its specific strengths and ideal use cases. The choice of tool should consider factors such as the type of data (e.g., 16S/18S rRNA or metagenomic data), analysis requirements (e.g., classification accuracy or processing speed), and available computational resources (Sempéré et al., 2021). Comprehensive platforms like QIIME 2 and MOTHUR are suitable for more integrated analyses, while tools like Kraken and MetaPhlAn are better for rapid classification and analysis of metagenomic data. BLAST and RDP are more suited for detailed sequence alignment and analysis of smaller datasets.

After sequencing and data processing, microbial abundance can be represented as a two-dimensional matrix count, where each value represents the estimated abundance of a taxon in a specific sample. Different bioinformatics analysis methods are then used for analysis and exploration. A common analysis pattern is to use software such as EdgeR to explore the relationship between microbial function and host phenotype by comparing the differential microbial abundance between the experimental and control groups (Robinson et al., 2010). With the enrichment of analytical methods, Bioconductor packages in R and software like Anaconda in Python have also been developed (Gentleman et al., 2004). Knight et al. elaborated on current methods of microbiome analysis in detail in their review (Knight et al., 2018).

### 2.2 Limitations of microbiome data analysis methods

In microbiome data analysis, there is often a low accuracy in species classification. In shotgun sequencing, abundance is calculated based on the counting of short reads (usually <300 bp) in the sequencing experiment, which are aligned to multiple reference genomes to determine their origin. Due to the large genetic variations and species diversity in the host’s microbiome, the short read sequences may not match any reference genome or may match multiple reference genomes (Tierney et al., 2019). Despite this limitation, researchers still use various methods for alignment and classification (Wood and Salzberg, 2014). In 16S rRNA amplicon sequencing, microorganisms are typically clustered and classified at defined thresholds, such as 97% or 99% sequence similarity, and the operational taxonomic units (OTUs) obtained from this classification process (Chiarello et al., 2022). However, due to limitations of sequencing technology, it results in a higher sequencing error rate. With technological advancements, methods such as amplicon sequence variants (ASV) or zero-radius operational taxonomic units (zOTUs) can now be used to cluster microorganisms more accurately. These methods not only enhance nucleotide resolution when resolving amplicons, but also use complex models to correct sequences that may contain errors (Amir et al., 2017; Antich et al., 2021; Chiarello et al., 2022).

In addition to the above limitations, due to the nature of microbial data as a set, the counts for a specific sample are relative abundance information compared to other taxa, rather than absolute counts (Gloor et al., 2017). Therefore, the subset representing the whole is constrained to 1 in the dataset (Gloor et al., 2016). To address this issue, although methods like additive or centered log-ratio transformations have been developed, caution should still be exercised in selecting statistical models for microbiome research to avoid drawing erroneous conclusions due to the relative abundance of taxa. Many taxa in the microbiome data of samples have zero counts. Zero counts may not necessarily reflect true biological signals (Chen et al., 2022). Therefore, this characteristic of microbiome data is also limiting the application of existing models, resulting in phenomena such as the “horse-shoe” pattern in dimensionality reduction methods like PCA and PCoA (Morton et al., 2017). Additionally, the gut microbiome is strongly influenced by factors such as changes over time, antibiotic use, and diet (Dudek-Wicher et al., 2018). A study by Vandeputte et al. found significant differences in the composition of the gut microbiome among different individuals (Vandeputte et al., 2021). Research by Johnson et al. indicates that the gut microbiota is significantly influenced by dietary factors (Johnson et al., 2019). In addition, microbiome data in animal models is also influenced by various factors such as cages, psychological stress, and the environment (Wang and LêCao, 2020). Therefore, in microbiome research, conclusions should not be drawn from short-term microbial measurements and analyses. Experiments should be conducted by increasing sample sizes and controlling dietary factors (Johnson et al., 2020).

### 2.3 Integrated multi-omics approach in microbiome studies

**Figure 1:**
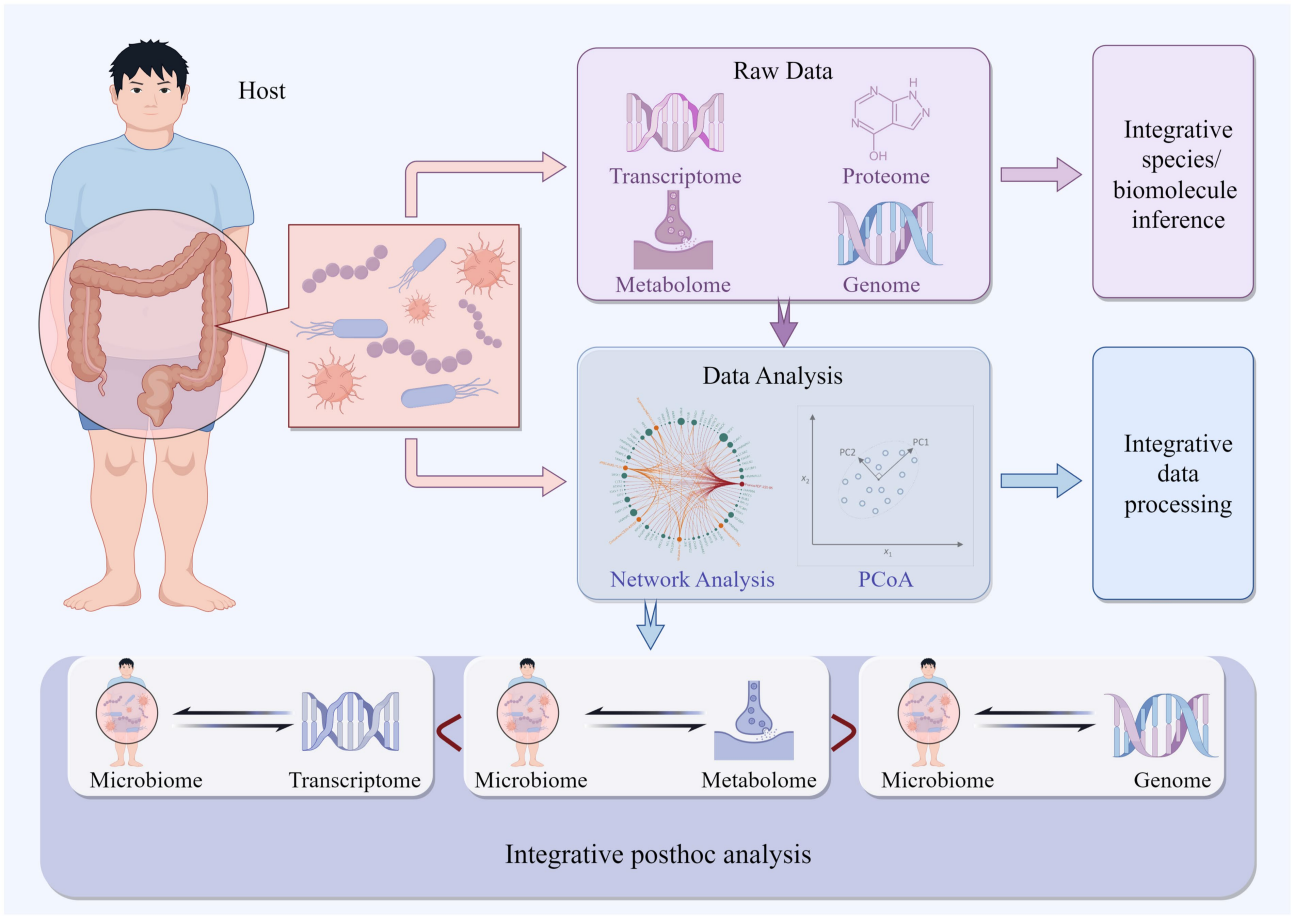
Depicts multi-omics integrated analysis occurring at various stages of the analysis process.

It is well known that multi-omics integrated analysis is beneficial for microbiome research. However, researchers have not yet reached a consensus on the best multi-omics integration method. Integration of multi-omics can occur at different stages of the analysis process, and researchers have proposed different multi-omics integration strategies for different research purposes (Figure 1). In some studies, the abundance of bacterial communities is estimated through the integration of metagenomics, metatranscriptomics, and proteomics from the beginning of the research (Heintz-Buschart et al., 2016b). In others, researchers introduce multi-omics data for batch correction during the data preprocessing process or integrated them during the data analysis process (Ugidos et al., 2022). More research strategies involve researchers conducting separate sequencing experiments for different omics, analyzing each omics dataset individually, and then integrating the results of each omics analysis (Forslund et al., 2021). This review summarizes various commonly used methods for microbiome association (Table 1).

**Table 1:** Principles and advantages of microbiome association methods.

Classification of methods	Method	Principle of analysis	Advantage	Study
Traditional Association Methods	Pearson	Reflect the degree of linear correlation between two continuous variables by the deviations of the two variables from their respective means	High test efficiency, strong interpretability	Pearson (1896)
	Spearman	Evaluate the correlation between two statistical variables using a monotonic equation	It does not require any specific data distribution and has a wide applicability range	Spearman (2015)
	SparCC	Fit the observed data to the Dirichlet distribution and calculate the correlation coefficient	Addressing the issue of excess negative correlations in traditional methods, known as the suppression of positive correlations	Friedman and Alm (2012)
	CCLasso	Use the least squares method with the L1 penalty term to infer compositional data, establish a model to infer the correlation between microorganisms	Accuracy and reproducibility are comparable to SparCC, but the error rate is better than SparCC	Fang et al. (2015)
Zero-inflated models	Zero-inflated negative binomial regression	Attributing zero observations in data to zeros generated by the data structure and sampling zeros	It is suitable for datasets with an excessive number of zero observations and has higher statistical efficiency	Lambert (1992)
Association method based on mutual information (MI)	KNN-MI	Find neighboring samples of the sample in the space formed by random variables X and Y, and calculate the mutual information	It does not require any specific data distribution and is suitable for nonlinear correlations	Kraskov et al. (2004)
	Kernel density MI	The method of kernel density estimation estimates the probability density function of continuous variables and calculates mutual information	It does not require any specific data distribution and is suitable for nonlinear correlations	Moon et al. (1995)
	MIC	Grid the scatter plot of variables in various ways and calculate the maximum mutual information value	Universal, fairness, and symmetry. Suitable for nonlinear correlations	Reshef et al. (2011)
Method for constructing interaction networks	mmvec	Estimate the co-occurrence probability of metabolites and microorganisms through neural network learning, and estimate the associative relationship	F1 score, precision, and recall are higher compared to traditional statistical methods	Morton et al. (2019)
	MIMOSA2	Compute the correlation by assessing the estimated metabolic capacity of the microbiome and the actual observed levels of metabolites	By integrating EGG or host information, more accurate results can be obtained.	Noecker et al. (2016)

Microbiome data analysis also faces issues such as sensitivity to analysis processes and excessive dependence on databases. In the process of bioinformatic analysis, when using different differential analysis software, different parameters, or aligning to different reference databases, researchers may obtain different results (Arora et al., 2020; Nearing et al., 2022; Peters et al., 2019). When conducting multi-omics integrated analysis, the impact caused by the analysis process may be amplified to a certain extent. Therefore, when studying or integrating data from multiple sources, researchers should ensure the impact of the analysis process is reduced by uniformly processing samples. Furthermore, multi-omics integrated analysis also relies on corresponding databases that support individual data analysis. For example, 16S rRNA amplicon sequencing requires microbial sequences to be aligned with known rRNA sequences stored in sequence databases such as SILVA (Quast et al., 2013). Similarly, metabolomics research may rely on databases such as Human Metabolome Database (HMDB) (Wishart et al., 2022). Transcriptomics research may rely on pathway databases, such as Kyoto Encyclopedia of Genes and Genomes (KEGG) (Kanehisa and Goto, 2000). Multi-omics integrated analysis means the need to introduce more databases. Due to the complexity of database construction, database updates are relatively slow. Therefore, the results of multi-omics integrated analysis based on microbiology may be affected by database updates, leading to significant differences in research results (Debelius et al., 2016; Nearing et al., 2022).

Dimensionality reduction analysis is typically the first step in any omics analysis, as it provides a rapid way to visualize the overall structure of a dataset. Commonly used dimensionality reduction methods include Principal Component Analysis (PCA), Principal Coordinate Analysis (PCoA), Isomap, t-SNE, and UMAP analysis. All dimensionality reduction methods perform different transformations to embed data into two-dimensional space, where PCA or PCoA typically constructs independent datasets for each sample before comprehensive analysis to identify sample distribution patterns (Silveira et al., 2021; Zhao et al., 2022). In addition to the above dimensionality reduction methods, methods such as Multi-omics Factor Analysis (MOFA) that can perform dimensionality reduction analysis on multi-omics data have also been proposed (Garcia-Etxebarria et al., 2021). Meng et al. reviewed various integrated dimensionality reduction analysis methods in their review (Meng et al., 2016). In addition to dimensionality reduction analysis methods, clustering algorithms are also commonly used to identify overall patterns in datasets. Common clustering methods include Euclidean distance, Manhattan distance, and Bray–Curtis dissimilarity. Currently, various clustering analysis methods have been used for clustering multi-omics datasets, enabling clustering analysis to more accurately capture the complex relationships between different omics. In 2007, Von Luxburg et al. proposed Spectral clustering and provided a detailed explanation of Spectral clustering in their paper (Luxburg, 2007).

The methods for determining the quantitative and covariate relationships of multi-omics data often require the computation of similarity metrics, such as Pearson correlation coefficient and Spearman correlation coefficient. The Pearson correlation cannot identify nonlinear relationships and is prone to discovering spurious correlations in the dataset. Although Spearman correlation can detect nonlinear relationships, it is also susceptible to finding spurious correlations in the dataset (Lovell et al., 2015). For the above reasons, methods utilizing similarity metrics such as Kendall’s tau (Liu et al., 2016), centered log ratio (CLR) (Gloor et al., 2016), SparCC (Friedman and Alm, 2012), REBACCA (Ban et al., 2015), mutual information (Tackmann et al., 2019), cosine similarity (Jackson et al., 2018), Canonical correlation analysis (CCA) (Sankaran and Holmes, 2019) and Procrustes analysis (Lisboa et al., 2014) have begun to be proposed. In a study, Faust et al. simultaneously used various methods such as Bray-Curtis, dissimilarity, Kullback–Leibler divergence, Pearson correlation, and Spearman correlation for correlation analysis (Faust et al., 2012). You et al. also compared multiple methods in a study of joint analysis of metabolomics and microbiome, and found that Spearman correlation was generally the most effective (You et al., 2019).

### 2.4 Multi-omics integration analysis based on the microbiome

With the advancement of technology, various omics technologies continue to emerge. In the multi-omics integration analysis based on the microbiome, in addition to the microbiome, a large amount of sequencing data from different omics, such as genomics, transcriptomics, proteomics, and metabolomics, are also analyzed. Depending on the different research subjects, there are certain differences in the research strategies of omics technologies (Table 2). Therefore, each multi-omics integration analysis model needs to consider factors that are essential, and we will review these considerations in this section.

**Table 2:** Single-omics techniques and their research methods.

Omics techniques	Research methods	Study
Microbiome	16 s sequencing, metagenomic sequencing, single-cell microbial sequencing	Knight et al. (2018)
Genomics	*De novo* sequencing, resequencing, simplified genome sequencing	Wang and Han (2022)
Metabolomics	Targeted metabolomics, untargeted metabolomics, lipidomics, spatial metabolomics	Liu and Locasale (2017)
Proteomics	Protein mass spectrometry identification, protein sequencing, quantitative proteome analysis, Post-translational modified proteomics, spatial proteomics, single-cell proteomics	González-Gomariz et al. (2019)
Transcriptomics	Translationomics, general transcriptome sequencing, single-cell transcriptome sequencing, spatial transcriptome sequencing technology, whole transcriptome sequencing, full-length transcriptome sequencing	Aldridge and Teichmann (2020)
Epigenomics	Genome-wide bisulfite sequencing, chromatin immunoprecipitation and sequencing, high-throughput chromosome capture, transposase-accessible chromatin sequencing, single-cell epigenome sequencing	Boix et al. (2021) and Preissl et al. (2023)

### 2.5 Microbiome and host genome joint analysis

Genome-wide association analysis (GWAS) is one of the most important methods for identifying genetic mutation sites in the host genome associated with gut microbiota. Currently, researchers have identified and distinguished a large number of SNP sites through GWAS, which can provide important clues for in-depth analysis of the genetic mechanisms of complex traits or diseases or open up new avenues. Therefore, drawing on the principles of traditional GWAS analysis of complex traits, researchers have proposed microbiome-wide association analysis (miGWAS) to explore the association between the host’s entire genome genetic markers and gut microbiota (Blekhman et al., 2015). However, due to the fact that gut microbiota data is not composed of simple multidimensional data, but rather a complex multidimensional trait. Individual microbial abundance data often exhibit uneven distribution, with many zero values and outliers present. Moreover, the complex biological interactions among microbiota lead to highly collinear relationships and complex structural correlations between microbial abundances (Kurilshikov et al., 2017). Currently, although there are many statistical analysis methods available for handling such complex data, there is still no single statistical method that is fully applicable to the interaction between host genetics and gut microbiota. The GWAS methods currently used are not entirely suitable for the localization analysis of microbiome quantitative trait loci (mbQTL).

For miGWAS analysis of gut microbiota, as shown in Table 3, there are currently two main methods widely used. One is based on traditional linear mixed models, which require phenotypes to follow a normal distribution. In early whole-genome mbQTL localization, phenotypes are typically transformed appropriately before applying linear mixed model GWAS analysis. For example, in mbQTL localization work conducted in mice, the FaST-LMM software is often used. Goodrich utilized the GEMMA software based on linear mixed models in a study of a twin cohort in the UK. Blekhman employed linear model methods used in plink (Kurilshikov et al., 2017). Due to suboptimal transformation effects of many microbiota abundance data, some studies have also employed statistical methods independent of traditional linear mixed models for the analysis of the association between host genetic variation and gut microbiota abundance. Wang et al. proposed that microbiota abundance data better fit a negative binomial distribution. Therefore, they used a generalized linear model conforming to the negative binomial distribution for statistical analysis. Additionally, for phenotypes with a substantial number of microbiota abundances being zero, they applied a hurdle model based on the negative binomial distribution for analysis. The hurdle model, also known as a two-part model, simultaneously considers the presence of microbiota and the relationship between microbiota abundance variations and host genetics. The first part of the model employs a binomial probability distribution model to determine the association between the presence of microbiota and the host genetic background (Xu et al., 2015). The second part of the model analyzes the portion of data where microbiota abundance is greater than 0 and its association with host genetic variation. In a similar vein to the two-part model, Turpin et al. utilized a model based on the generalized estimating equations for a log-normal model (Turpin et al., 2016). Furthermore, Bonder et al. employed the Spearman rank-sum test method to conduct an association analysis between gut microbiota abundance and host genetic variation (Bonder et al., 2016).

**Table 3:** Summary of the analysis protocols for miGWAS.

Sequencing method	Traits	Model	Study
16Sseq	Beta-diversity	Envfit: ordinaon-based, permutan test for significance	Wang J. et al. (2016)
16Sseq	Beta-diversity	Microbiome GWAS: distance-based, parametric	Goodrich J. et al. (2016)
16Sseq; WGS	Enterotype	Enterotype GWAS: logistic model implemented in PLINK	Liu et al. (2021)
16Sseq; WGS	Bacterial taxa	Combined two-part logit/lognormal model	Turpin et al. (2016)
16Sseq; WGS	Bacterial taxa	GEMMA: Genomewide efficient mixed models	Goodrich J. et al. (2016)
16Sseq; WGS	Bacterial taxa	Hurdle negative binomial model	Wang J. et al. (2016)
16Sseq; WGS	Bacterial taxa	Spearman correlaon excluding zero incidence	Bonder et al. (2016)
WGS	Bacterial pathways	Spearman correlaon excluding zero incidence	Bonder et al. (2016)

Additionally, we know from extensive research on microbiota, particularly human gut microbiota (Li et al., 2014; Qin et al., 2010), that the number of microbial genes in the gut far exceeds those of the host and plays a central role in metabolism and immune regulation (Clemente et al., 2012; Marchesi et al., 2016). Therefore, Qin et al. from BGI-Shenzhen introduced the concept and methodology of Metagenome-Wide Association Study (MGWAS) for the first time in 2012, using GWAS as a model. They conducted MGWAS analysis based on deep shotgun sequencing of gut microbial DNA from 345 Chinese individuals (Qin et al., 2012). Their study identified 60,000 molecular markers associated with type 2 diabetes. MGWAS analysis revealed that patients with type 2 diabetes exhibit moderate gut microbiota dysbiosis and a reduced abundance of butyrate-producing microorganisms. In general, MGWAS not only can identify changes at a high-resolution strain level but also can identify enriched or decreased microbial functions based on annotations from databases such as KEGG, COG, and EggNOG in diseased individuals. In addition to type 2 diabetes and obesity, MGWAS has also been used in the research of human diseases such as colorectal cancer (Zeller et al., 2014) and rheumatoid arthritis (Zhang X. et al., 2015). With the advancement in the field of microbiology, MGWAS is expected to have broader applications in studying the influence of gut microbiota on host complex traits.

### 2.6 Microbiome and metabolome joint analysis

In omics technologies, metabolomics plays a crucial role in linking host phenotypes and microbial functional profiles (Fiehn, 2002; Patti et al., 2012). Metabolomics is a systematic study of all small molecules within a biological system. Unlike other omics, metabolites and metabolic pathways are relatively conserved across species. The gut metabolome includes metabolites produced by both the host and the microbial community. Conducting a joint analysis of the microbiome and metabolome helps in understanding the interactions between gut microbial functions and the host. In recent years, with technological advancements, a plethora of bioinformatics tools and analytical methods have been developed for single omics analyses (Dhariwal et al., 2017; Xia et al., 2012; Xia et al., 2009). However, methods for multi-omics joint analysis are still relatively scarce (Gautam et al., 2023; Ni et al., 2020). The key point in the joint analysis of the microbiome and metabolome lies in the integration of multi-omics data. Microbiome and metabolome data consist of two or more matrices that share sample IDs but contain different biological variables, such as metabolites or operational taxonomic units (OTUs). Currently, two main methods of data integration are used to combine microbiome and metabolome data. (1) Statistical integration: Utilizing univariate or multivariate analyses to understand the correlations between biological variables in different omics datasets; (2) Knowledge-driven integration: By projecting important biological variables identified from individual omics onto existing knowledge bases to understand potential mechanistic links, thereby constructing interaction networks.

The simplest method in statistical integration is univariate correlation analysis, which aims to determine whether there is a strong linear relationship (Pearson correlation) or a monotonic relationship (Spearman correlation) between individual metabolites (metabolome) and taxonomic groups (microbiome). For example, in a multi-omics study of the goat rumen microbiome, Mao et al. used univariate correlation methods to establish a Pearson correlation matrix between genera and metabolites (Mao et al., 2016). The authors found a clear correlation between changes in rumen microbial community structure and metabolite profiles with increasing carbohydrate intake (Mao et al., 2016). While univariate correlation analysis is relatively straightforward, these methods have a higher false positive rate, leading to lower reliability of research results. While multivariate methods are more complex than univariate methods, they allow for the simultaneous consideration of interactions between data matrices and within data matrices, significantly increasing the reliability of the analysis results. On the other hand, due to the high-dimensional nature of omics data, dimensionality reduction methods have become a primary approach for statistical integration. The purpose of dimensionality reduction techniques is to reduce a large number of variables to a small number of new components or principal variables with minimal information loss. For example, El Aidy et al. (2013) used O2-PLS to integrate pairwise the metabolomic, transcriptomic, and metagenomic data of germ-free mice colonized with the gut microbiota of normal mice. The authors found a strong correlation between early microbial colonizers and changes in urine metabolites, as well as a correlation between colonic tissue metabolites and upregulation of genes involved in O- and N-glycan biosynthesis and degradation (El Aidy et al., 2013). Canonical correlation analysis (CCA) (Moser et al., 2018) and co-inertia analysis (CIA) (Thioulouse and Lobry, 1995) are two other commonly used multivariate correlation methods in omics integration. CCA is a feature extraction method that identifies the optimal linear combinations of X and Y to maximize the correlation between the components. Co-inertia analysis (CIA) was initially used in ecological studies and later applied to omics integration. It describes the shared structure between two datasets by maximizing the covariance between components. CIA first applies data reduction techniques such as PCA or correspondence analysis to X and Y separately, then constrains the generated components to maximize the squared covariance between X and Y (Thioulouse and Lobry, 1995).

Knowledge-driven omics integration methods leverage existing knowledge frameworks about relationships between metabolites, species, and/or genes to integrate different omics data. This information can be gathered through literature mining or computationally predicted from public databases. The simplest form of knowledge-based omics integration is through association networks, which are created based on pairwise relationships between biological entities measured in omics data. Pairwise relationships can be computed directly from omics data itself or based on third-party resources. For instance, McHardy et al. (2013) constructed interaction networks of the cecum and colon based on pairwise Spearman correlations between microbiome and metabolome data. While correlation-based network reconstructions involve interactions between microbial species, they do not provide more detailed mechanistic information about these interactions. Metabolic models, comprehensive reconstructions of an organism’s metabolism, serve as an alternative to the interaction-based network methods used previously. These models can serve as a scaffold for integrating omics data, thereby providing crucial mechanistic details about microbial community functions and activities.

### 2.7 Microbiome and metaproteome joint analysis

Given that gut microbes constitute over 90% of the total microbial population in the host, current metaproteomic research is predominantly centered on gut microbiota. Samples collected from the host gut contain the microbiome and host proteins. These microbiome/host proteins directly represent the functional activities of the gut ecosystem. Macroproteomic analysis can quantify the proteins produced by the host and microbiome, providing a basis for a deeper understanding of the functional roles of microbes in host health (Peters et al., 2019). As a complement to metagenomics and metatranscriptomics, macroproteomic analysis reflects the activity of cellular translation and post-translational processes. Similar to metabolomics, macroproteomics is typically achieved through mass spectrometry analysis. One advantage of macroproteomics over metabolomics is the ability to obtain information on sample classification and functional activities. When functional variations are observed, this information enables researchers to assess the contributions specific to phylogenetic development. In the context of microbiome and metaproteome joint analysis, the use of macroproteomics to assess microbial functions has been shown to be superior to 16S rRNA gene sequencing (Kleiner et al., 2017), further highlighting the value of metaproteomics in microbiome research. Studies have shown that with sufficient depth of macroproteomic measurements, macroproteomics can also be used to analyze abundance information of microbial communities (Xiong et al., 2017).

As metagenomics has been widely used in microbiome research, a high proportion of previous studies on gut microbiome using macroproteomics or metabolomics have been conducted through metagenomics. By integrating shotgun metagenomics with macroproteomics, not only can protein expression levels be quantified, but protein identification can also be achieved by generating matched sample metagenomic databases. Using a matched shotgun metagenomic database search approach, Mills RH et al. conducted an integrated metagenomic/macroproteomic study of the microbiome in patients with Crohn’s disease, revealing consistent changes in genes, proteins, and pathways compared to the control group (Mills et al., 2019). In healthy adults, Tanca et al.’s study found that the taxonomic composition of microbial communities obtained using metagenomics and macroproteomics is generally comparable. However, metagenomics (representing functional potential) and macroproteomics (representing functional activity) exhibit significant differences, with macroproteomics showing higher inter-individual variability (Tanca et al., 2017). Heintz-Buschart et al. conducted a more integrated multi-omics study of the microbiome in type 1 diabetes (T1DM) patients, providing a good example of integrated multi-omics data integration (Heintz-Buschart et al., 2016a). In summary, metagenomic and metatranscriptomic data are first utilized for co-assembling the genome and predicting microbial genes in the gut. The latter are then translated into protein sequences and used for protein identification in metaproteomics. This integrated data processing workflow enables efficient integration of all three omics datasets and assesses the relationships between microbial proteins or functions encoded, transcribed, and expressed.

### 2.8 Joint analysis of the microbiome with other omics data

In current research methodologies, the integration of multi-omics (including phylogenetic marker-based microbiome analysis, shotgun metagenomics, metatranscriptomics, metaproteomics, metabolomics, genetic variations, gene expression, and epigenetics) is one of the important approaches to reveal the interactions between host genetics and microbial communities by combining diverse data from both the host and microbes, providing new insights into microbial functional studies. Through host transcriptomics, researchers can quantify gene expression activities under different treatment or disease states, thereby gaining insights into the interactions between host genes and the microbiome (Conesa et al., 2016). Analysis techniques in metatranscriptomics enable researchers to quantify the abundance of microbial gene transcripts in samples, aiding in a deeper understanding of microbial functional characteristics. The research protocols in metatranscriptomics vary depending on the organism under study. For instance, after next-generation sequencing, transcripts are aligned to a metatranscriptomic reference genome for quantitative analysis (Shakya et al., 2019).

Currently, research methods for integrating multi-omics analysis can be broadly categorized into two main types: one common approach involves fixing host genetics, such as using twin cohorts (Goodrich J.K. et al., 2016) or genetically modified animals (Carmody et al., 2015) as subjects to study the interactions between host genetics and gut microbiota. This method significantly reduces the workload of collecting host genotypes; however, it is limited to individual genes or genes previously reported, making it challenging to generate new hypotheses about host–microbe interaction mechanisms. The other approach directly correlates host genomic variation data, gene expression data, epigenetic information, etc., with gut metagenomic data, metatranscriptomic data, and even metaproteomic data. By integrating high-dimensional host information data with high-dimensional microbial data, correlations between the host and gut microbiota can be discovered statistically. This integrative approach of multi-omics data plays an increasingly important role in microbiome research. For example, several studies have identified associations between host genomic variations and gut microbiota (Blekhman et al., 2015; Goodrich J. et al., 2016), with some findings validated in multiple populations (Turpin et al., 2016).

Although integrating multi-omics poses greater statistical challenges for researchers, such as the use of efficient bioinformatics tools and advanced statistical methods (multivariate statistics and machine learning methods) (Blanco-Míguez et al., 2019; Knight et al., 2018; Mallick et al., 2017; Valles-Colomer et al., 2016), this integration of high-dimensional host data and microbial data analysis is playing an increasingly important role in research. However, since factors like environment, diet, and ecological factors can also influence the composition of the gut microbiota among individuals, and may be related to host genetics (Knights et al., 2014). Therefore, it is crucial to control these factors through experimental or statistical methods. As host genetic information can also predict gene expression in specific tissues, in the future, integrating host genotypes and microbiome information may help investigate the expression interaction network between the host and the microbiome.

## 3 Based on the progress of multi-omics integrated analysis of the microbiome

### 3.1 Progress in the joint analysis of the microbiome and host genome

In human studies, the initial research on the joint analysis of the microbiome and host genome was conducted with candidate genes set. Researchers found several significant associations between the microbiome and host genetics in the context of candidate genes. Early studies revealed associations between the Fucosyltransferase 2 (*FUT2*) gene and microbial energy metabolism and mucosal inflammation (Tong et al., 2014), as well as between the *MEFV* gene and changes in bacterial phylum abundance (Khachatryan et al., 2008). Furthermore, a study conducted the first human whole-genome mbQTL mapping in 93 individuals with both metagenomic and genotype data within the Human Microbiome Project, indicating a correlation between the two (Blekhman et al., 2015). Subsequently, researchers carried out three independent large-scale population studies on mbQTL. Bonder et al. (2016), Turpin et al. (2016), and Wang et al. J. (2016) conducted high-resolution QTL mapping in populations from the Netherlands, Canada, and Germany, respectively. All three groups used similar experimental designs in fairly large cohorts and found similar results (Table 4).

**Table 4:** Summary of the combined analysis of microbiome and host genome.

Study	Sequencing method	Analysis	Population sample size	Number of Gene/Loci	Organism
Frank et al. (2011)	16S seq	Gene	178	1	Humans
Blekhman et al. (2015)	WGS	Gene	93	1	Humans
Davenport et al. (2015)	16S seq	Gene	184	1	Humans
Bonder et al. (2016)	WGS	Gene	1,514	2	Humans
Turpin et al. (2016)	16S seq	Gene	1,561	9	Humans
Lim et al. (2017)	16S seq	Gene	655	1	Humans
Xie et al. (2016)	16S seq	Gene	1,126	8	Humans
Hughes et al. (2020)	16S seq	Gene	3,890	11	Humans
Ishida et al. (2020)	16S seq	Gene	1,068	5	Humans
Kurilshikov et al. (2021)	16S seq	Gene	18,340	9	Humans
Benson et al. (2010)	16Sseq	QTL	645	18	Mouse
Hillhouse et al. (2011)	16Sseq	QTL	314	10	Mouse
McKnite et al. (2012)	16Sseq	QTL	61	9	Mouse
Leamy et al. (2014)	16Sseq	QTL	472	42	Mouse
Org et al. (2015)	16Sseq	QTL	599	7	Mouse
Wang J. et al. (2016)	16Sseq	QTL	334	20	Mouse
Snijders et al. (2016)	16Sseq	QTL	293	169	Mouse
Kemis et al. (2019)	16Sseq	QTL	500	28	Mouse
Perez-Munoz et al. (2019)	16Sseq	QTL	128	27	Mouse
Suzuki et al. (2004)	16Sseq	QTL	70	24	Mouse
Bubier et al. (2020)	16Sseq	QTL	500	18	Mouse
Zhao et al. (2013)	16Sseq	Heritability	60	13	Chicken
Chen et al. (2017)	16Sseq	Heritability	500	74	Pig
Wen et al. (2021)	16Sseq	Heritability	206	47	Chicken
Fan et al. (2021)	16Sseq	Heritability	278	9	Bovine
Wang et al. (2022)	16Sseq	Heritability	239	NA	Pig

In addition, research on the joint analysis of the microbiome and host genome is not limited to humans. Due to the complexity of human populations and ethical considerations, some studies have also been conducted in experimental animals (Table 4). As the most common experimental animals, Benson et al. (2010) conducted a miGWAS study in mice and identified 26 mbQTLs associated with the abundance of 64 microbial taxa. Some of these mbQTLs exhibit pleiotropy, where multiple different genetic loci influence one or more microbial traits. It is worth noting that regardless of whether the microbial taxa are correlated, they may be regulated by the same genetic loci. For example, a study found that an mbQTL on chromosome 7 affected two phylogenetically close bacteria while an mbQTL on chromosome 10 affected taxonomically unrelated lactobacilli and coriobacteriaceae. Subsequently, researchers conducted functional predictions on these selected mbQTLs that affect microbial abundance and found that many of the mbQTLs’ functions may be related to host obesity, immunity, and disease susceptibility.

These studies have all confirmed the interactions between the host genome and the composition of the microbiome, identifying the pleiotropy of relevant loci. They have also highlighted several host phenotype-associated loci that have genetic effects on the microbiome composition. Furthermore, due to the high similarity in genetic microbiota and functional categorization of candidate genes among pigs, chickens, cattle, and mice, it suggests that the genetic effects of the host on the gut microbiota of different mammals are similar. This enhances researchers’ comprehensive and in-depth understanding of the interplay between the microbiome and host genome.

### 3.2 The progress of research on the joint analysis of the microbiome and metabolome

With the advancement of technology, metabolomics has become a powerful tool for studying individual metabolic differences in health and disease. Analyzing the fecal metabolome of individuals with inflammatory bowel disease (IBD) and colorectal cancer (CRC) revealed significant changes in the fecal metabolome of diseased individuals compared to healthy individuals.

In a study by Jansson et al., researchers used untargeted metabolomics analysis to identify the contributions of metabolites produced by the gut microbiota to the host’s disease state. Ion Cyclotron Resonance Fourier Transform Mass Spectrometry (ICR-FT/MS) was used to discern the masses of thousands of metabolites in fecal samples collected from 17 identical twin pairs, including healthy individuals and those with CD. Pathways with differentiating metabolites included those involved in the metabolism and or synthesis of amino acids, fatty acids, bile acids and arachidonic acid. Several metabolites were positively or negatively correlated to the disease phenotype and to specific microbes previously characterized in the same samples (Jansson et al., 2009). Furthermore, Jacobs et al. studied the pre-disease risk status of inflammatory bowel disease (IBD) in first-degree relatives of 21 children with IBD. The results indicate individuals were classified into 2 microbial community types. One was associated with IBD but irrespective of disease status, had lower microbial diversity, and characteristic shifts in microbial composition including increased Enterobacteriaceae, consistent with dysbiosis. This microbial community type was associated similarly with IBD and reduced microbial diversity in an independent pediatric cohort. Individuals also clustered bioinformatically into two subsets with shared fecal metabolomics signatures. One metabotype was associated with IBD and was characterized by increased bile acids, taurine, and tryptophan. The IBD-associated microbial and metabolomics states were highly correlated, suggesting that they represented an integrated ecosystem (Jacobs et al., 2016). Franzosa et al. performed untargeted metabolomic and shotgun metagenomic profiling of cross-sectional stool samples from discovery (*n* = 155) and validation (*n* = 65) cohorts of CD, UC and non-IBD control patients. Metabolomic and metagenomic profiles were broadly correlated with fecal calprotectin levels (a measure of gut inflammation). Across >8,000 measured metabolite features, they identified chemicals and chemical classes that were differentially abundant in IBD, including enrichments for sphingolipids and bile acids, and depletions for triacylglycerols and tetrapyrroles (Franzosa et al., 2019). In addition, in recent years, a large number of studies involving multi-omics integrative analysis of the microbiome and metabolome have been conducted (Table 5). Through the joint analysis of the microbiome and metabolome, researchers have further elucidated how metabolites change with different physiological states in the complex life system of the host.

**Table 5:** Summary of the combined analysis of microbiome and Metabolome.

Study	Years	Metabolomic technique	Sample type	Population sample size	Disease
Marchesi et al. (2007)	2007	^1^H NMR	Fecal water	33 samples	IBD (CD and UC)
Jansson et al. (2009)	2009	ICR-FT-MS	Fecal water	34 samples	IBD (CD)
Phua et al. (2014)	2014	GC-TOFMS	Fecal sample	21 samples	Colorectal cancer (CRC)
Zhang C. et al. (2015)	2015	1H NMR	Fecal water and urine sample	38 samples	Prader–Willi syndrome (PWS) and simple obesity
Jacobs et al. (2016)	2016	UPLC-MS	Fecal sample	90 samples	IBD (CD and UC)
Sinha et al. (2016)	2016	LC/GC–MS	Fecal sample	132 samples	CRC
Wang et al. (2017)	2017	GC–MS	Fecal sample	27 samples	CRC
Franzosa et al. (2019)	2019	LC–MS	Fecal sample	220 sample	IBD (CD and UC)

### 3.3 Progress in research on the joint analysis of the microbiome and the proteome

Although metaproteomics is still in its early stages and new technologies are under development, research on metaproteomics has begun and has provided a new perspective on the functionality of the microbiome from another level. It has offered new insights into the various physiological processes involved in health and disease states. Metaproteomics has been used to analyze the gut microbiota of patients with complex diseases such as IBD (Juste et al., 2014) and cirrhosis (Wei et al., 2016). As shown in Table 6, these studies have been able to more accurately identify microbial differences in experimental samples by comparing metaproteomic data from healthy and diseased individuals. Furthermore, changes in microbial metabolic pathways and alterations in host–microbe interaction networks can be further observed, aiding in elucidating the role of the gut microbiome in various diseases.

**Table 6:** Summary of the combined analysis of microbiome and metaproteomics.

Study	Sequencing method	Sample type	Population sample size	Number of identified protein groups	Disease
Juste et al. (2014)	16Sseq	Fecal sample	12 samples	Not reported	IBD (CD)
Erickson et al. (2012)	mNGS	Fecal sample	12 samples	2,904 (healthy), 1,928 (ileal CD), 2,241 (colonic CD) on sample average	IBD (CD)
Michail et al. (2015)	16Sseq	Fecal sample	50 samples	96 (healthy), 104 (NAFLD) on sample average	Non-alcoholic fatty liver disease (NAFLD)
Kolmeder et al. (2015)	Metaproteomics	Fecal sample	29 samples	Not reported	Obesity
Wei et al. (2016)	Metaproteomics	Fecal sample	6 samples	5,020	Liver cirrhosis
Li et al. (2016)	Metaproteomics	MLI lavage	51 samples	Not reported	IBD (CD and UC)
Gavin et al. (2018)	Metaproteomics	Fecal sample	101 samples	11,378	Type 1 Diabetes (T1DM)
Chen et al. (2018)	Metaproteomics	Fecal sample	20 samples	2,440	Major depressive disorder (MDD)
Zhang et al. (2020)	Metaproteomics	MLI lavage	176 samples	53,207	IBD (CD and UC)
Blakeley-Ruiz et al. (2019)	Metaproteomics	Fecal sample	25 samples	14,850	IBD (CD) in remission

Catherine and colleagues conducted a study on the IBD population. They first developed and validated a workflow-including extraction of microbial communities, two-dimensional difference gel electrophoresis (2D-DIGE), and LC–MS/MS-to discover protein signals from CD-associated gut microbial communities. Then they used selected reaction monitoring (SRM) to confirm a set of candidates. In parallel, they used 16S rRNA gene sequencing for an integrated analysis of gut ecosystem structure and functions. Their 2D-DIGE-based discovery approach revealed an imbalance of intestinal bacterial functions in CD. Many proteins, largely derived from Bacteroides species, were over-represented, while under-represented proteins were mostly from Firmicutes and some Prevotella members. Moreover, although the abundance of most protein groups reflected that of related bacterial populations, they found a specific independent regulation of bacteria-derived cell envelope proteins (Juste et al., 2014). Michail and colleagues conducted another study on the population with non-alcoholic fatty liver disease (NAFLD), and the study found that, children with NAFLD had more abundant Gammaproteobacteria and Prevotella and significantly higher levels of ethanol, with differential effects on short chain fatty acids. This group also had increased genomic and protein abundance for energy production with a reduction in carbohydrate and amino acid metabolism and urea cycle and urea transport systems. The metaproteome and metagenome showed similar findings. The gut microbiome in pediatric NAFLD is distinct from lean healthy children with more alcohol production and pathways allocated to energy metabolism over carbohydrate and amino acid metabolism, which would contribute to development of disease (Michail et al., 2015).

Metaproteomics has not only been applied to study gut microbiota but also to investigate microbial communities from other sources, such as the human oral microbiome (Jersie-Christensen et al., 2018), vaginal microbiome (Berard et al., 2018), as well as environmental microbial communities in water (Hettich et al., 2012) and sediment ecosystems (Wang D.Z. et al., 2016), allowing for a deeper understanding of the functions of these microbial communities. While significant differences between sample types require different sample collection and preprocessing procedures, and distinct microbial compositions necessitate specialized microbial databases for better identification, it is encouraging that mass spectrometry techniques, databases, and functional analysis methods have already begun to be applied despite the variations among biological samples.

## 4 Conclusion and future directions

In this review, we have summarized the multi-omics integrative analysis methods based on the microbiome and briefly outlined their initial applications. The characteristic of multi-omics technologies is the organic integration of information from various omics dimensions, constructing gene regulatory networks, comprehensively exploring and deeply understanding the regulatory and causal relationships among various biological molecules, thereby correctly deciphering the biological functions and physiological mechanisms of organisms. The strategy of multi-omics integrative analysis is to normalize, compare, and correlate batch data from different omics levels for specific biological functions in the same integrated analysis software, establishing correlations between molecular data at different levels. Simultaneously, combining GO functional analysis, metabolic pathway enrichment, molecular interactions, and other biological functional analysis systems comprehensively elucidates the functions and regulatory mechanisms of biological molecules. The application of multi-omics integrative analysis can further clarify the complex relationships among various biological molecules involved in the host, microbiome, and their interactions, providing new insights into disease biology.

An emerging application of multi-omics analysis is in precision medicine. In precision medicine research, measurement data from multiple omics levels are used to guide and formulate treatment plans tailored to the specific physiological state of patients. Due to the multifactorial effects of the microbiome, it can provide a promising target for precision medicine. For example, adjusting drugs or doses based on a patient’s microbiome composition or other molecular phenotypes may benefit disease treatment. Although various methods have been developed for multi-omics integrative analysis, the lack of standardization and other issues can lead to research results being prone to false positives. Therefore, there is an urgent need at this stage to establish an optimal approach for integrating multi-omics data, which will help to gain a more in-depth and specific understanding of the role of the microbiome in host biological processes.
